# Southern Ocean Asteroidea: a proposed update for the Register of Antarctic Marine Species

**DOI:** 10.3897/BDJ.3.e7062

**Published:** 2015-11-25

**Authors:** Camille VE Moreau, Antonio Aguera, Quentin Jossart, Bruno Danis

**Affiliations:** ‡ Université Libre de Bruxelles (ULB), 50 avenue F. Roosevelt 1050, Brussels, Belgium

**Keywords:** Asteroidea, Sea stars, Southern Ocean, RAMS, WoRMS, Register of Antarctic Marine Species, Biodiversity, Checklist

## Abstract

**Background:**

The Register of Antarctic Marine Species (RAMS, De Broyer et al. 2015) is the regional component of the World Register of Marine Species (WoRMS Editorial Board 2015) in the Southern Ocean. It has been operating for the last ten years, with a special effort devoted towards its completion after the International Polar Year (IPY) in 2007-2008, in the framework of the Census of Antarctic Marine Life (CAML, 2005 - 2010). Its objective is to offer free and open access to a complete register of all known species living in the Southern Ocean, building a workbench of the present taxonomic knowledge for that region. The Antarctic zone defined by this dynamic and community-based tool has been investigated with a particular interest. The Sub-Antarctic zone was a secondary objective during the establishment of the RAMS and is still lacking the impulse of the scientific community for some taxa.

**New information:**

In the present study, more than 13,000 occurrences records of Asteroidea (Echinodermata) have been compiled within the RAMS area of interest and checked against the RAMS species list of sea stars, using WoRMS Taxon Match tool. Few mismatches (basionym mistakes : i.e. original name misspelled or incorrect) were found within the existing list and 97 unregistered species are actually occurring within the RAMS boundaries. After this update, the number of Asteroidea species was increased by around 50%, now reaching 295 accepted species.

## Introduction

### The Register of Antarctic Marine Species, RAMS

RAMS is a collaborative and dynamic information system managing Southern Ocean marine taxon names and related information (De Broyer et al. 2015). The main objective of RAMS is to establish a benchmark of the present taxonomic knowledge of the Southern Ocean biodiversity (De Broyer et al. 2011).

In a recent appraisal of RAMS, Jossart et al. (2015)​ underscored the number of 10,294 described species of which 8,297 are accepted marine species (*viz.* checked by taxonomic experts). Among these species, 537 (~6.5%) were reported as accepted species names for echinoderms and 198 (~2.4%) as accepted names for sea star species.

After 10 years of service, RAMS still displays several spatial gaps, especially with regards to species occurring in the sub-Antarctic zone, described as the area below the sub-Tropical front (Deacon 1984, Rintoul 2007) and North of the Polar Front.

In order to complete the taxonomic information for the class Asteroidea, we propose in this work an updated version of the checklist of Southern Ocean sea stars species occurring within the RAMS area of interest.

## Materials and methods

### Area of interest

**Figure 1.**
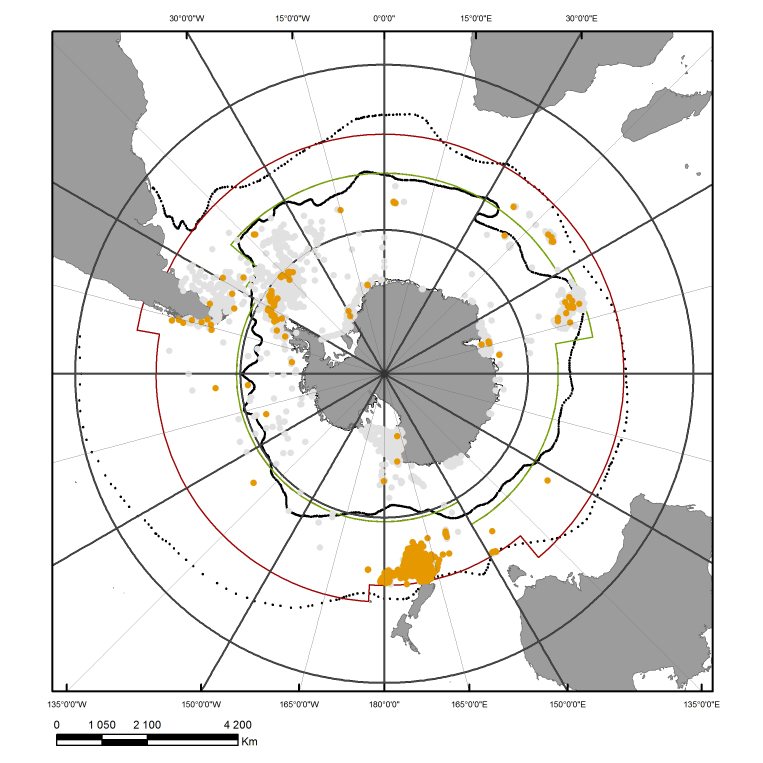
Occurrences of RAMS (light grey dots) and Proposed-RAMS (orange dots) sea star species. The Antarctic zone is located below the green line and the Sub-Antarctic zone between the green and dark red lines. The Polar Front (black line) and the Sub-Tropical Front (dashed black line) are also shown.

As described in De Broyer and Danis (2011) the RAMS area of interest is extending from the coast of the Antarctic continent to the sub-Tropical front. Convenient operational limits have been defined (Table 1; Fig. 1), splitting the Southern Ocean into two zones: the Antarctic zone and the sub-Antarctic zone.

### Data collection

More than 13,000 occurrences records were agregated from different sources including global information systems such as the Ocean Biogeographic Information System (OBIS), the Global Biodiversity Information Facility (GBIF), initiatives such as the Biogeographic Atlas of the Southern Ocean (De Broyer et al. 2014), historic records mined from the literature pertaining to the early exploration of the Southern Ocean (e.g. Sladen 1889), recently published checklists (e.g. Gutt et al. 2014) or unpublished cruise records. Only the specimens identified at a species level, regardless of their depth (shelf, slope, deep-sea)  were kept for the preparation of this checklist.

The validity of each species name was controlled using the Taxa Match Tool available in RAMS (http://www.marinespecies.org/rams/aphia.php?p=match) and WoRMS (http://www.marinespecies.org/aphia.php?p=match) to ensure its validity and presence in the RAMS checklist.

## Data resources

### Complete checklist of Southern Ocean Asteroidea

The data underpinning the analyses reported in this paper are deposited at GBIF, the Global Biodiversity Information Facility, http://ipt.pensoft.net/resource?r=southern_ocean_asteroidea&v=1.1

## Checklists

### Checklist of RAMS Asteroidea species

#### Abyssaster diadematus

(Sladen, 1883)

#### Abyssaster planus

(Sladen, 1883)

#### Acodontaster capitatus

(Koehler, 1912)

#### Acodontaster conspicuus

(Koehler, 1920)

#### Acodontaster elongatus

(Sladen, 1889)

#### Acodontaster hodgsoni

(Bell, 1908)

#### Acodontaster marginatus

(Koehler, 1912)

#### Adelasterias papillosa

(Koehler, 1906)

#### Allostichaster capensis

(Perrier, 1875)

#### Anasterias antarctica

(Lütken, 1857)

#### Anasterias asterinoides

Perrier, 1875

#### Anasterias directa

(Koehler, 1920)

#### Anasterias mawsoni

(Koehler, 1920)

#### Anasterias pedicellaris

Koehler, 1923

#### Anasterias perrieri

(E. A. Smith, 1876)

#### Anasterias rupicola

(Verrill, 1876)

#### Anasterias sphoerulata

(Koehler, 1920)

#### Anasterias spirabilis

(Bell, 1881)

#### Anasterias studeri

Perrier, 1891

#### Anasterias suteri

(deLoriol, 1894)

#### Anseropoda antarctica

Fisher, 1940

#### Anteliaster australis

Fisher, 1940

#### Anteliaster scaber

(E. A. Smith, 1876)

#### Asterina fimbriata

Perrier, 1875

#### Astropecten brasiliensis

Müller & Troschel, 1842

#### Bathybiaster loripes

Sladen, 1889

#### Belgicella racowitzana

Ludwig, 1903

#### Benthopecten pedicifer

(Sladen, 1885)

#### Caimanaster acutatus

Clark, 1962

#### Calyptraster tenuissimus

Bernasconi, 1966

#### Calyptraster vitreus

Bernasconi, 1972

#### Ceramaster grenadensis

(Perrier, 1881)

#### Ceramaster patagonicus

(Sladen, 1889)

#### Cheiraster (Luidiaster) antarcticus

(Koehler, 1907)

#### Cheiraster (Luidiaster) gerlachei

Ludwig, 1903

#### Cheiraster (Luidiaster) hirsutus

(Studer, 1884)

#### Cheiraster (Luidiaster) planeta

(Sladen, 1889)

#### Chitonaster cataphractus

Sladen, 1889

#### Chitonaster felli

(H.E.S. Clark, 1971)

#### Chitonaster johannae

Koehler, 1908

#### Chondraster elattosis

H.L. Clark, 1923

#### Cladaster analogus

Fisher, 1940

#### Cosmasterias lurida

(Philippi, 1858)

#### Crossaster penicillatus

Sladen, 1889

#### Cryptasterias brachiata

Koehler, 1923

#### Cryptasterias turqueti

(Koehler, 1906)

#### Ctenodiscus australis

Lütken, 1871

#### Ctenodiscus procurator

Sladen, 1889

#### Cuenotaster involutus

(Koehler, 1912)

#### Cycethra frigida

(Koehler, 1917)

#### Cycethra macquariensis

Koehler, 1920

#### Cycethra verrucosa

(Philippi, 1857)

#### Diplasterias brandti

(Bell, 1881)

#### Diplasterias brucei

(Koehler, 1908)

#### Diplasterias kerguelenensis

(Koehler, 1917)

#### Diplasterias meridionalis

(Perrier, 1875)

#### Diplasterias octoradiata

(Studer, 1885)

#### Diplasterias radiata

(Koehler, 1923)

#### Diplodontias singularis

(Müller & Troschel, 1843)

#### Diplopteraster clarki

Bernasconi, 1937

#### Diplopteraster peregrinator

(Sladen, 1882)

#### Diplopteraster semireticulatus

(Sladen, 1882)

#### Diplopteraster verrucosus

(Sladen, 1882)

#### Dytaster felix

Koehler, 1907

#### Echinaster smithi

Ludwig, 1903

#### Eremicaster crassus

(Sladen, 1883)

#### Eremicaster pacificus

(Ludwig, 1905)

#### Eremicaster vicinus

Ludwig, 1907

#### Freyastera tuberculata

(Sladen, 1889)

#### Freyella attenuata

Sladen, 1889

#### Freyella drygalskii

Döderlein, 1927

#### Freyella formosa

Korovchinsky, 1976

#### Freyella fragilissima

Sladen, 1889

#### Freyella giardi

Koehler, 1908

#### Freyella heroina

Sladen, 1889

#### Freyella mutabila

Korovchinsky, 1976

#### Ganeria attenuata

Koehler, 1907

#### Ganeria falklandica

Gray, 1847

#### Ganeria hahni

Perrier, 1891

#### Gaussaster antarcticus

(Sladen, 1889)

#### Glabraster antarctica

(E. A. Smith, 1876)

#### Granaster nutrix

(Studer, 1885)

#### Henricia diffidens

(Koehler, 1923)

#### Henricia fisheri

A.M. Clark, 1962

#### Henricia obesa

(Sladen, 1889)

#### Henricia pagenstecheri

(Studer, 1885)

#### Henricia parva

Koehler, 1912

#### Henricia praestans

(Sladen, 1889)

#### Henricia smilax

(Koehler, 1920)

#### Henricia studeri

Perrier, 1891

#### Hippasteria falklandica

Fisher, 1940

#### Hippasteria phrygiana

(Parelius, 1768)

#### Hymenaster caelatus

Sladen, 1882

#### Hymenaster campanulatus

Koehler, 1908

#### Hymenaster coccinatus

Sladen, 1882

#### Hymenaster crucifer

Sladen, 1882

#### Hymenaster densus

Koehler, 1908

#### Hymenaster edax

Koehler, 1908

#### Hymenaster formosus

Sladen, 1882

#### Hymenaster fucatus

Koehler, 1908

#### Hymenaster graniferus

Sladen, 1882

#### Hymenaster latebrosus

Sladen, 1882

#### Hymenaster pellucidus

Thomson, 1873

#### Hymenaster perspicuus

Ludwig, 1903

#### Hymenaster praecoquis

Sladen, 1882

#### Hymenaster sacculatus

Sladen, 1882

#### Hymenodiscus distincta

(Sladen, 1889)

#### Hyphalaster inermis

Sladen, 1883

#### Hyphalaster scotiae

Koehler, 1907

#### Kampylaster incurvatus

Koehler, 1920

#### Kenrickaster pedicellaris

A.M. Clark, 1962

#### Labidiaster annulatus

Sladen, 1889

#### Labidiaster radiosus

Lütken, 1872

#### Leptychaster flexuosus

(Koehler, 1920)

#### Leptychaster kerguelenensis

E. A. Smith, 1876

#### Leptychaster magnificus

(Koehler, 1912)

#### Leptychaster melchiorensis

(Bernasconi, 1969)

#### Lethasterias australis

Fisher, 1923

#### Lonchotaster tartareus

Sladen, 1889

#### Lophaster densus

Fisher, 1940

#### Lophaster gaini

Koehler, 1912

#### Lophaster stellans

Sladen, 1889

#### Lophaster tenuis

Koehler, 1920

#### Luidia clathrata

(Say, 1825)

#### Lysasterias adeliae

(Koehler, 1920)

#### Lysasterias belgicae

(Ludwig, 1903)

#### Lysasterias chirophora

(Ludwig, 1903)

#### Lysasterias digitata

A.M. Clark, 1962

#### Lysasterias hemiora

Fisher, 1940

#### Lysasterias heteractis

Fisher, 1940

#### Lysasterias joffrei

(Koehler, 1920)

#### Lysasterias lactea

(Ludwig, 1903)

#### Lysasterias perrieri

(Studer, 1885)

#### Macroptychaster accrescens

(Koehler, 1920)

#### Mediaster pedicellaris

(Perrier, 1881)

#### Mirastrella biradialis

Fisher, 1940

#### Neosmilaster georgianus

(Studer, 1885)

#### Neosmilaster steineni

(Studer, 1885)

#### Notasterias armata

(Koehler, 1911)

#### Notasterias bongraini

(Koehler, 1912)

#### Notasterias candicans

(Ludwig, 1903)

#### Notasterias haswelli

Koehler, 1920

#### Notasterias pedicellaris

(Koehler, 1907)

#### Notasterias stolophora

Fisher, 1940

#### Notioceramus anomalus

Fisher, 1940

#### Novodinia novaezelandiae

(H.E.S. Clark, 1962)

#### Odinella nutrix

Fisher, 1940

#### Odontaster meridionalis

(E. A. Smith, 1876)

#### Odontaster penicillatus

(Philippi, 1870)

#### Odontaster pusillus

Koehler, 1907

#### Odontaster validus

Koehler, 1906

#### Paralophaster antarcticus

(Koehler, 1912)

#### Paralophaster godfroyi

(Koehler, 1912)

#### Paralophaster lorioli

(Koehler, 1907)

#### Pectinaster filholi

Perrier, 1885

#### Pedicellaster hypernotius

Sladen, 1889

#### Pergamaster incertus

(Bell, 1908)

#### Pergamaster triseriatus

H.E.S. Clark, 1963

#### Peribolaster folliculatus

Sladen, 1889

#### Peribolaster macleani

Koehler, 1920

#### Perknaster antarcticus

(Koehler, 1906)

#### Perknaster aurantiacus

Koehler, 1912

#### Perknaster aurorae

(Koehler, 1920)

#### Perknaster charcoti

(Koehler, 1912)

#### Perknaster densus

Sladen, 1889

#### Perknaster fuscus

Sladen, 1889

#### Perknaster sladeni

(Perrier, 1891)

#### Persephonaster facetus

(Koehler, 1907)

#### Poraniopsis echinaster

Perrier, 1891

#### Porcellanaster ceruleus

Wyville Thomson, 1877

#### Psalidaster mordax

Fisher, 1940

#### Pseudarchaster discus

Sladen, 1889

#### Psilaster charcoti

(Koehler, 1906)

#### Pteraster affinis

Smith, 1876

#### Pteraster florifer

Koehler, 1920

#### Pteraster gibber

(Sladen, 1882)

#### Pteraster hirsutus

(Sladen, 1882)

#### Pteraster koehleri

A.M. Clark, 1962

#### Pteraster rugatus

Sladen, 1882

#### Pteraster spinosissimus

(Sladen, 1882)

#### Pteraster stellifer

Sladen, 1882

#### Radiaster gracilis

(H.L. Clark, 1916)

#### Remaster gourdoni

Koehler, 1912

#### Rhopiella hirsuta

(Koehler, 1920)

#### Saliasterias brachiata

Koehler, 1920

#### Scotiaster inornatus

Koehler, 1907

#### Smilasterias clarkailsa

O'Loughlin & O'Hara, 1990

#### Smilasterias scalprifera

(Sladen, 1889)

#### Smilasterias triremis

(Sladen, 1889)

#### Solaster notophrynus

Downey, 1971

#### Solaster regularis

Sladen, 1889

#### Styracaster armatus

Sladen, 1883

#### Styracaster chuni

Ludwig, 1907

#### Styracaster horridus

Sladen, 1883

#### Styracaster robustus

Koehler, 1908

#### Tremaster mirabilis

Verrill, 1880

#### Vemaster sudatlanticus

Bernasconi, 1965

#### Zoroaster tenuis

Sladen, 1889

### Checklist of Proposed-RAMS Asteroidea species

#### Allostichaster farquhari

McKnight, 2006

#### Allostichaster insignis

(Farquhar, 1895)

#### Allostichaster polyplax

(Muller & Troschel, 1844)

#### Anasterias laevigata

(Hutton, 1879)

#### Anthenoides cristatus

(Sladen, 1889)

#### Astromesites primigenius

(Mortensen, 1925)

#### Astropecten polyacanthus

Müller & Troschel, 1842

#### Astrostole scabra

(Hutton, 1872)

#### Benthopecten munidae

H.E.S. Clark, 1969

#### Benthopecten pikei

H.E.S. Clark, 1969

#### Brisinga chathamica

McKnight, 1973

#### Brisingenes multicostata

(Verrill, 1894)

#### Ceramaster australis

H.E.S. Clark, 2001

#### Cheiraster (Cheiraster) otagoensis

Studer, 1883

#### Chitonaster trangae

Mah, 2011

#### Clavaporania fitchorum

Mah & Foltz, 2014

#### Coscinasterias calamaria

(Gray, 1840)

#### Coscinasterias muricata

Verrill, 1870

#### Cosmasterias dyscrita

H.L. Clark, 1916

#### Crossaster campbellicus

McKnight, 1973

#### Crossaster multispinus

H.L. Clark, 1916

#### Diplodontias dilatatus

(Perrier, 1875)

#### Diplodontias robustus

(Fell, 1953)

#### Diplopteraster hurleyi

McKnight, 1973

#### Dipsacaster magnificus

(H.L. Clark, 1916)

#### Echinaster farquhari

Benham, 1909

#### Eratosaster jenae

Mah, 2011

#### Freyastera benthophila

(Sladen, 1889)

#### Freyella echinata

Sladen, 1889

#### Freyellaster polycnema

(Sladen, 1889)

#### Fromia monilis

(Perrier, 1869)

#### Gilbertaster anacanthus

Fisher, 1906

#### Henricia aucklandiae

Mortensen, 1925

#### Henricia compacta

(Sladen, 1889)

#### Henricia lukinsii

(Farquhar, 1898)

#### Henricia ornata

(Perrier, 1869)

#### Henricia ralphae

Fell, 1958

#### Henricia simplex

(Sladen, 1889)

#### Henricia spinulfera

(E. A. Smith, 1876)

#### Hymenaster estcourti

McKnight, 1973

#### Hymenaster nobilis

Wyville Thomson, 1876

#### Hymenodiscus aotearoa

(McKnight, 1973)

#### Hymenodiscus submembranacea

(Döderlein, 1927)

#### Hyphalaster giganteus

Macan, 1938

#### Lithosoma novaezelandiae

McKnight, 1973

#### Luidia porteri

A.H. Clark, 1917

#### Mediaster arcuatus

(Sladen, 1889)

#### Mediaster dawsoni

McKnight, 1973

#### Mediaster sladeni

Benham, 1909

#### Meridiastra medius

(O'Loughlin, Waters & Roy, 2003)

#### Meridiastra oriens

(O'Loughlin, Waters & Roy, 2003)

#### Mimastrella cognata

(Sladen, 1889)

#### Myxoderma qawashqari

(Moyana & Larrain Prat, 1976)

#### Odontaster aucklandensis

McKnight, 1973

#### Odontaster benhami

(Mortensen, 1925)

#### Odontaster pearsei

Janosik & Halanych, 2010

#### Odontaster roseus

Janosik & Halanych, 2010

#### Odontohenricia anarea

O'Hara, 1998

#### Odontohenricia endeavouri

Rowe & Albertson, 1988

#### Ophidiaster confertus

H.L. Clark, 1916

#### Paralophaster hyalinus

H.E.S. Clark, 1970

#### Paranepanthia aucklandensis

(Koehler, 1920)

#### Patiriella regularis

(Verrill, 1867)

#### Paulasterias tyleri

Mah et al. 2015

#### Pectinaster mimicus

(Sladen, 1889)

#### Pentagonaster pulchellus

Gray, 1840

#### Peribolaster lictor

Fell, 1958

#### Perissasterias monacantha

McKnight, 1973

#### Pillsburiaster aoteanus

(McKnight, 1973)

#### Pillsburiaster indutilis

McKnight, 2006

#### Plutonaster complexus

H.E.S Clark & D.G. McKnight, 2000

#### Plutonaster fragilis

H.E.S. Clark, 1970

#### Plutonaster hikurangi

H.E.S Clark & D.G. McKnight, 2000

#### Plutonaster jonathani

H.E.S Clark & D.G. McKnight, 2000

#### Plutonaster knoxi

Fell, 1958

#### Plutonaster sirius

A.H. Clark, 1917

#### Proserpinaster neozelanicus

(Mortensen, 1925)

#### Psalidaster fisheri

McKnight, 2006

#### Pseudarchaster garricki

Fell, 1958

#### Pseudechinaster rubens

H.E.S. Clark, 1962

#### Psilaster acuminatus

Sladen, 1889

#### Pteraster bathami

Fell, 1958

#### Pteraster robertsoni

McKnight, 1973

#### Sclerasterias eustyla

(Sladen, 1889)

#### Sclerasterias mollis

(Hutton, 1872)

#### Smilasterias irregularis

H.L. Clark, 1928

#### Solaster longoi

Stampanato & Jangoux, 1993

#### Solaster torulatus

Sladen, 1889

#### Sphaeriodiscus mirabilis

A.M. Clark, 1976

#### Stichaster australis

(Verrill, 1871)

#### Taranuiaster novaezealandiae

McKnight, 1973

#### Tarsaster stoichodes

Sladen, 1889

#### Zoroaster actinocles

Fisher, 1919

#### Zoroaster alternicanthus

McKnight, 2006

#### Zoroaster fulgens

Thomson, 1873

#### Zoroaster macracantha

H.L. Clark, 1916

#### Zoroaster spinulosus

Fisher, 1906

## Analysis

More than 13,000 occurrences records from 295 accepted species have been compiled within the extent of the RAMS area of interest. 198 of these species (67%) were already in the RAMS database and 97 (33%) were new to the system. After this update, the number of Asteroidea species in RAMS will be increased by around 50%.

The seven Orders of the Class Asteroidea are represented in the Southern Ocean. The Valvatida are the most speciose with 8 families, 41 genus and 87 species followed by Forcipulatida (6 families, 30 genus, 78 species), Paxillosida (6 families, 22 genus, 43 species), Velatida (2 families, 6 genus, 37 species), Spinulosida (1 family, 4 genus, 20 species), Brisingida (2 families; 9 genus; 19 species) and Notomyotida (1 family, 4 genus, 2 subgenus, 11 species).

After a careful verification of the RAMS species list for Asteroidea we propose to address the following points. These issues have been taken into account in this paper:

- Cheiraster (Barbadosaster) echinulatus and *Stegnaster wesseli* are only described from Central America and should be removed from the RAMS list
- *Spoladaster veneris* is only described from Amsterdam and Saint-Paul Islands which are not part of the RAMS area of interest
- *Anthenoides peircei*, *Astropecten cingulatus*, Cheiraster (Cheiraster) sepitus, *Peltaster placenta*, and *Psilaster herwigi* do not seem to be distributed in the RAMS area of interest
- Echinaster (Othilia) brasiliensis is only described from South America and should be removed of the RAMS list​
- *Freyella mutabilia* was originally described as *Freyella mutabila* by Korovchinsky (1976)
- *Mimastrella cognata* and *Mirastrella cognata* are source of a problem. Both genus do exist but *Mimastrella cognata* and *Mirastrella biradialis* are the only described species. The genus name being nearly identical, it has lead to a mistake. *Mirastrella cognata* is an invalid name and should be deleted from RAMS and WoRMS systems

Moreover, we recommend the addition of species from our "Proposed-RAMS" checklist (i.e. species not registered in the RAMS list but present in the RAMS area of interest) to RAMS. None of these species are new to science but their austral distribution range was not properly documented (e.g. species from the Campbell Plateau are reported as New-Zealand species but are also present in the Southern Ocean, species from the French sub-Antarctic Islands, etc...). Interestingly, 22 of the 97 "non-RAMS" species are present in the Antarctic zone. Some have been described recently (e.g. *O. roseus* and *O. pearsei* described by Janosik and Halanych (2010); *C. trangae* described by Mah (2011) or *P. tyleri* described by Mah et al. 2015) but have not been added to the RAMS.

A final comment pertains to *Astropecten polyacanthus*, occurring in the RAMS Sub-Antarctic region in a location that is not actually South from the Sub-Tropical Front. This final observation shows that convenient boundaries should be used with care. However, only one species was concerned for a very wide area of interest. *A. polyacanthus* presence in the RAMS checklist should be discussed by the editors from RAMS and WoRMS.

## Discussion

This study highlights the fact that after ten years of effort, the work is still in progress for RAMS. The main objective of covering the Antarctic zone has generally been reached for the class Asteroidea but needs at this point to go through a major update by the editors. Regarding the asteroids, the secondary objective of covering the Sub-Antarctic zone lacked the impulse of the scientific community and we hope that the work presented here will fill the gaps as accurately as possible.

This approach may not bring the same results for all the taxa in the Southern Ocean. Indeed some charismatic fauna are very well known in the RAMS area of interest (e.g. marine mammals), however, we believe that the knowledge concerning the number of species present in the Southern Ocean waters might be significantly increased.

Only few mistakes were found in the existing RAMS list of Asteroidea and should be fixed soon after getting in contact with the editors. The new updated checklist of Asteroidea species will be available through the RAMS website (http://www.marinespecies.org/rams) in early 2016. A way to improve the general system, with the sea stars for model is also in development.

Recent work using genetics on *Odontaster* species (Janosik and Halanych 2010) highlights the fact that diversity might be higher than expected even in well-studied areas. There is also a lack of ressources for identification and taxonomic work leading to redundant mistakes. In this context, the creation of a digital library, hotlinked with the RAMS and compiling original descriptions, literature materials and DNA barcoding informations for each species will bring the RAMS a step ahead and provide a vital tool for future taxonomic and biogeographic work. Perspectives also include an illustrative determination key using a polytomic approach and the creation of interactive Antarctic Field Guides (http://afg.biodiversity.aq) on the http://www.biodiversity.aq platform (Van de Putte et al. 2015) in order to help the scientific community with the identification process and to enhance data availability.

We believe that the homogenization of available data for all taxa will result in a better understanding of the Southern Ocean and its biogeography, especially in the Sub-Antarctic zone.

## Figures and Tables

**Table 1.** RAMS area of interest. Operational northern limits in the different sector of the Southern Ocean for both the Antarctic zone and the sub-Antarctic zone.

**Sector**	**Longitude range**	**Northern limit**
**Antarctic zone**		
South Atlantic Sector	60°W-50°W	57°S
	50°W-30°E	50°S
Indian Sector	30°E-80°E	50°S
	80°E-150°E	55°S
South Pacific Sector	150°E-60°W	60°S
**Sub-Antarctic zone**		
Atlantic & Indian Sectors	60°W-140°E	43°S
South Pacific Sector	140°E-176°W	48°S
	176°W-80°W	45°S
	80°W-72°W	41°S
